# Large investment of stored nitrogen and phosphorus in female cones is consistent with infrequent reproduction events of *Pinus koraiensis*, a high value woody oil crop in Northeast Asia

**DOI:** 10.3389/fpls.2022.1084043

**Published:** 2023-01-12

**Authors:** Haibo Wu, Jianying Zhang, Jesús Rodríguez-Calcerrada, Roberto L. Salomón, Dongsheng Yin, Peng Zhang, Hailong Shen

**Affiliations:** ^1^ State Key Laboratory of Tree Genetics and Breeding, School of Forestry, Northeast Forestry University, Harbin, China; ^2^ Department of Natural Systems and Resources, Universidad Politécnica de Madrid. Ciudad Universitaria s/n, Madrid, Spain; ^3^ Key Laboratory of Sustainable Forest Ecosystem Management-Ministry of Education, Northeast Forestry University, Harbin, China; ^4^ State Forestry and Grassland Administration Engineering Technology Research Center of Korean Pine, Harbin, China; ^5^ Forestry Research Institute of Heilongjiang Province, Harbin, China

**Keywords:** Korean pine, strobili, branch growth, nutrient concentration, seasonal dynamics

## Abstract

*Pinus koraiensis* is famous for its high-quality timber production all the way and is much more famous for its high value health-care nut oil production potential since 1990’s, but the less understanding of its reproduction biology seriously hindered its nut productivity increase. Exploring the effects of reproduction on nutrient uptake, allocation and storage help to understand and modify reproduction patterns in masting species and high nut yield cultivar selection and breeding. Here, we compared seasonality in growth and in nitrogen ([N]) and phosphorus ([P]) concentrations in needles, branches and cones of reproductive (cone-bearing) and vegetative branches (having no cones) of *P. koraiensis* during a masting year. The growth of one- and two-year-old reproductive branches was significantly higher than that of vegetative branches. Needle, phloem and xylem [N] and [P] were lower in reproductive branches than in vegetative branches, although the extent and significance of the differences between branch types varied across dates. [N] and [P] in most tissues were high in spring, decreased during summer, and then recovered by the end of the growing season. Overall, [N] and [P] were highest in needles, lowest in the xylem and intermediate in the phloem. More than half of the N (73.5%) and P (51.6%) content in reproductive branches were allocated to cones. There was a positive correlation between cone number and N and P content in needles (R^2^ = 0.64, R^2^ = 0.73) and twigs (R^2^ = 0.65, R^2^ = 0.62) of two-year-old reproductive branches. High nutrient sink strength of cones and vegetative tissues of reproductive branches suggested that customized fertilization practices can help improve crop yield in *Pinus koraiensis*.

## 1 Introduction

Many tree species undergo significant variations in seed production from year-to-year, a phenomenon known as masting (Kelly, 1994; Kelly and Sork, 2002; Pearse et al., 2016; Allen et al., 2017; Fernández-Martínez et al., 2019; Fernández-Martínez et al., 2020; Kelly, 2020). Inter-annual variations in seed production have been related to climatic conditions (Allen et al., 2014; Roland et al., 2014; Pearse et al., 2016; Fernández-Martínez et al., 2019; LaMontagne et al., 2020), which affect annual growth (Yasumura et al., 2006; Smith and Samach, 2013; Nakahata et al., 2021), flowering (Law et al., 2000; Cook et al., 2012), pollen availability and pollination efficiency (Koenig and Knops, 2005; Koenig et Al., 2012; Pérez-Ramos et al., 2015; Pearse et al., 2016; Venner et al., 2016). In addition to climatic conditions, nutrient cycling is essential in regulating masting behaviour and reproductive mechanisms (Kelly, 1994; Kelly and Sork, 2002; Sala et al., 2012; Pearse et al., 2016; Han et al., 2017; Fernández-Martínez et al., 2019; Fernández-Martínez et al., 2020; Kelly, 2020) because reproduction consumes a significant amount of carbohydrates and mineral nutrients. Experimental evidence suggests that masting species accumulate reserves during 2-4 years for the subsequent masting reproduction event to occur (Sork et al., 1993; Yamauchi, 1996; Satake and Bjørnstad, 2008; Pearse et al., 2016). During the masting event, a significant fraction of resources is allocated to reproduction (e.g. fruit and seed development) to the detriment of growth or defense (Venner et al., 2016; Allen et al., 2017; Nakahata et al., 2021).

Korean pine (*Pinus koraiensis* S. et Z.) is a monoecious evergreen gymnosperm that naturally distributed in Northeast China, Korean Peninsula and Russia Far-east (Shen, 2003; Wang and Chen, 2004). *P. koraiensis* is a major source of timber and edible pine nuts due to its excellent wood properties and the substantial production of nutritious seeds and trees reach reproductive age at 20 - 30 years old, and female cones take two years to develop. (Shen, 2003; Wang and Chen, 2004; Xie et al., 2016; Zhang et al., 2017; Liao et al., 2021). Like many other pine species, *P. koraiensis* is a prominent masting tree, and its inter-annual periodicity is 3 to 5 years (Shen, 2003; Cheng et al., 2017), with massive cone production during the masting year consuming a large share of carbohydrates and mineral nutrients (Han et al., 2017; Yin et al., 2019; Wu et al., 2021). *P. koraiensis* is one of the 4 major nut trees globally (Xie et al., 2016); however, compared with other orchard trees (e.g. almond or chestnut), *P. koraiensis* remains at an early stage of domestication. There is certain blindness in the management and fertilization regimes of the species, which significantly limits its economic potential.

Mineral nutrients required for plant growth, development, and reproduction are mainly taken up from the soil by the roots and transported upwards through the xylem to organs aboveground (Wiley and Helliker, 2012; Congreves, et al., 2021). Nevertheless, mineral nutrients consumed for spring growth are commonly remobilized from storage tissues rather than taken up by the roots (Han et al., 2008; Nowak-Dyjeta et al., 2017), as water and nutrient transport through the xylem is still constrained by a low evaporative demand (Millard and Grelet, 2010; El Zein et al., 2011), as observed in *Picea* and *Pseudotsuga* seedlings (Van den Driessche, 1985; Proe and Millard, 1994). The development of reproductive organs constitutes an additional resource sink that competes with growth and storage. Nitrogen (N) and phosphorus (P) are two macronutrients often limiting the growth and reproduction of masting plant species (Newbery et al., 2006; Sala et al., 2012; Ichie and Nakagawa, 2013; Han et al., 2014) which can be readily translocated from leaves and woody tissues to reproductive organs for seed maturation (Miyazaki et al., 2002; Han et al., 2011). Likewise, vegetative branches neighboring reproductive branches can also act as nutrient suppliers (Munoz et al., 1993; Miyazaki et al., 2014), a frequent behavior during masting events (Sork et al., 1993; Satake and Bjørnstad, 2008; Ichie and Nakagawa, 2013; Sala et al., 2012; Miyazaki et al., 2014). Therefore, N and P content commonly decreases during the masting event throughout tree organs (Ichie and Nakagawa, 2013; Sala et al., 2012; Miyazaki et al., 2014). Nevertheless, nutrient allocation patterns seem to be species-, organ- and nutrient-specific. For example, sduring the masting of *Pinus albicaulis*, N and P concentrations ([N] and [P] hereafter) were reduced compared to previous, non-masting years only in reproductive branches, while during the following year, [N] and [P] depletion occurred in both vegetative and reproductive branches (Sala et al., 2012). In branches, stem and roots of *Dryobalanops aromatica*, [P] was reduced by more than half during reproduction compared to a non-masting year, while [N] remained stable (Ichie and Nakagawa, 2013). Therefore, a better understanding of mineral nutrient tree demand, absorption capacity and allocation during reproduction cycles will help design species-specific fertilization treatments, with the ultimate goal of shortening reproduction cycles and increasing gross seed production in the long term. In line with this, previous studies in different species have suggested that N- or P-fertilization enhances tree growth (Turner et al., 2002; Jasim, 2013), improves pollen, ovule viability and seed production (Callahan et al., 2008; Smaill et al., 2011; Ghanem et al., 2014; Bogdziewicz et al., 2017), and reduces the interval of masting events (Bogdziewicz, 2022).

This study aims to assess whether the growth of reproductive (cone-bearing) branches is inhibited by the mast event and whether cone maturation depletes nutrient availability in reproductive branches of Korean pine. To answer these questions, we monitored the seasonal dynamics of stored nutrients and the reproductive output (cone yield) in Korean pine during a masting year. Specifically, [N] and [P] seasonality in needles, twigs and cones of reproductive and vegetative branches were measured. We also examined the effect of masting on the growth of young (one- and two-year-old) branches. We hypothesize that (i) the growth of young reproductive branches will be lower than that of vegetative branches due to the diversion of nutrients and carbohydrates for reproductive purposes. Likewise, we predict (ii) stronger N and P depletion during cone maturation in vegetative tissues (needle, phloem and xylem) of reproductive branches relative to vegetative ones. We further expect (iii) the reproductive output to be inversely related to [N] and [P] in vegetative tissues at the seasonal timescale.

## 2 Materials and methods

### 2.1 Study site and sampled trees

This study was conducted at the Maoershan Research Station of the Northeast Forestry University (127°30′-127°34′E, 45°21′-45°25′N; Heilongjiang, China), in the northwest ridge of the Zhangguangcai Mountains. The area is characterized by a continental temperate monsoon climate, with warm, humid summers and cold, dry winters. The mean annual temperature is 2.8°C, and the mean temperatures in the coldest (January) and hottest (July) months are -19.6°C and 20.9°C, respectively. The growing season lasts from May to September, approximately 120 to 140 days. The mean annual precipitation is 723 mm, with 477 mm occurring from June to August. Soils are Hap-Boric Luvisols, with high organic matter content and good drainage. More details on the site and soil characteristics can be found in Wang et al. (2006). This study was conducted during the 2018 growing season in a 5-ha *P. koraiensis* stand planted in 1968. The site is located 490-510 m above sea level and has an average slope of 15° facing the north. The mean (± standard error) height of the trees in 2018 was 13.5 (± 0.6) m, and the mean diameter at breast height was 34.0 (± 3.6) cm.

### 2.2 Sampling time and protocol

**Figure 1:**
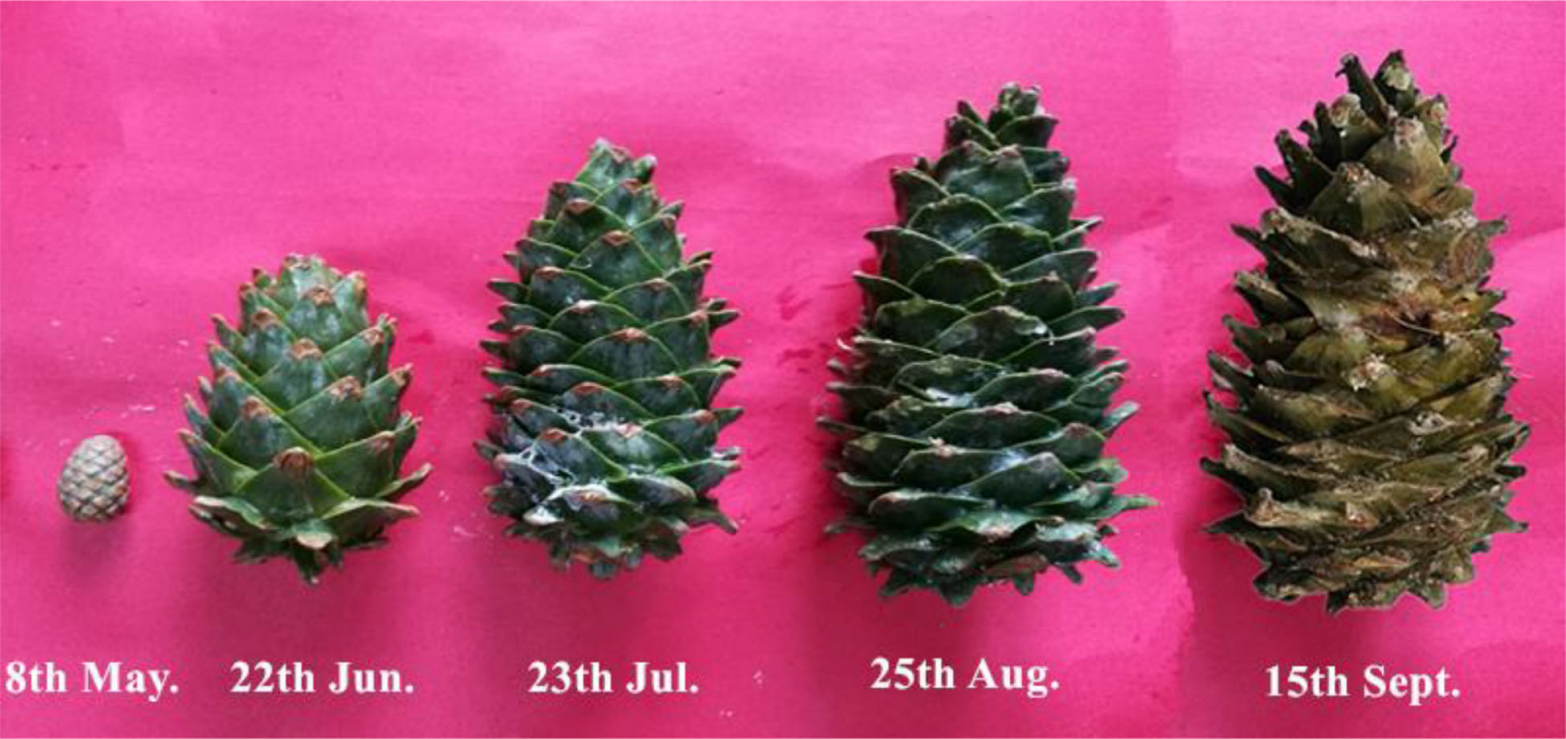
Female cones of *Pinus koraiensis* at different developmental stages. The numbers below each cone denote the sampling dates at May, June, July, August and September, corresponding to DOYs 128, 172, 204, 237 and 258, respectively.

Five healthy trees with a large production of cones accessible for climbing were selected for measurements. Trees were sufficiently spaced to prevent significant shading by neighbors. For each tree, we selected five branches bearing cones (i.e. reproductive branches) and five branches with no cones (i.e. vegetative branches) from the sun-lit southwest section of the upper canopy. Selected reproductive and vegetative branches were spaced at least 2 m to avoid the influence of cones on traits of vegetative branches. One reproductive and one vegetative branch per tree were harvested five times during the 2018 growing season. In the fifth sampling time, two additional reproductive branches were sampled to better analyze the relation between cone number and end-of season nutrient status. Sampling dates were established according to the development of female cones (hereafter, cones; **Figure 1**): (1) before the appearance of new shoots and cone growth (May; DOYs (day of year) 128); (2) at an early stage of cone growth, when new shoots had begun to grow (June; DOYs 172); (3) at a stage of rapid cone expansion, when new shoots had stopped growing (July; DOYs 204); (4) when the cones are ripe and ready for harvesting (August; DOYs 237); (5) when the cones are fully mature, and trees are nearly dormant (September; DOYs 258).

At each sampling time, the branches were transported to the laboratory with an ice cooler. In the laboratory, the length and width of cones were measured. Foliar (needles), xylem, phloem (including bark) and cone (including seeds) tissues were manually separated to determine their dry biomass. For this, organs were oven-dried in a forced-air oven at 75°C until constant dry mass. The dried material was ground into a fine powder in a steel ball mill (Retsch MM400, German) and dry-stored until further biochemical analyses. In the last sampling date (DOYs 258), diameter and length were measured in one- and two-year-old branches.

### 2.3 N and P concentrations

We used an automatic Kjeldahl analyzer (model KT260; Foss Inc., Hillerod, Denmark) to determine the total [N]. A subsample of each tissue was digested in 98% H_2_SO_4_ (w/w) and 30% H_2_O_2_, using CuSO_4_ and K_2_SO_4_ as catalysts to transform N into (NH_4_)_2_SO_4_. Thereafter, a 40% NaOH solution (w/v) was used to release NH_3_ from (NH_4_)_2_SO_4_. Finally, 1% H_3_BO_3_ was used to transform the released NH_3_ into (NH_4_)_2_B_4_O_7_. A solution of 0.1 M HCl was used to titrate the content of (NH_4_)_2_B_4_O_7._

We used a modified H_2_O_2_-H_2_SO_4_ method to determine the total [P] (Rapp et al., 1999; Shen et al., 2019). We digested 0.2 g of ground material in 5 mL of 98% H_2_SO_4_ and 2 mL of 30% H_2_O_2_ at 400°C for 2-3 h. When the solution had reached 100°C, 30% H_2_O_2_ was added dropwise until the solution became pale yellow or colorless. The digests were diluted, filtered through Whatman 2 filter paper, and finally topped up to 50 mL with deionized water. The concentration of P in the solution was determined at 700 nm with a spectrophotometer (UV-PC01; Shimadzu Corp., Kyoto, Japan). The content of N and P in each tissue was estimated by multiplying [N] and [P] by the corresponding dry biomass of each tissue.

### 2.4 Statistical analysis

Linear mixed models were adjusted per surveyed dependent variable. These include biomass, [N], [P], N content, and P content in needles, phloem and xylem of one- and two-year-old twigs. Branch type (i.e. vegetative vs reproductive), harvest time and their interaction were treated as fixed factors, while the tree was considered a random factor. When significant (*P* < 0.05), *post-hoc* LSD tests were applied for multiple comparisons. One-way analysis of variance (ANOVA) was used to evaluate the effect of harvest time on cone width, length, [N], [P], N content, and P content in reproductive branches. Finally, linear regressions between cone number and N and P content were adjusted separately for needle and twig tissues (xylem and phloem) for reproductive branches. Statistical analyses were performed using SPSS 26.0 for Windows (SPSS, Chicago, USA), and figures were plotted with SigmaPlot 10.0 (Systat Software, San Jose, USA).

## 3 Results

**Figure 2:**
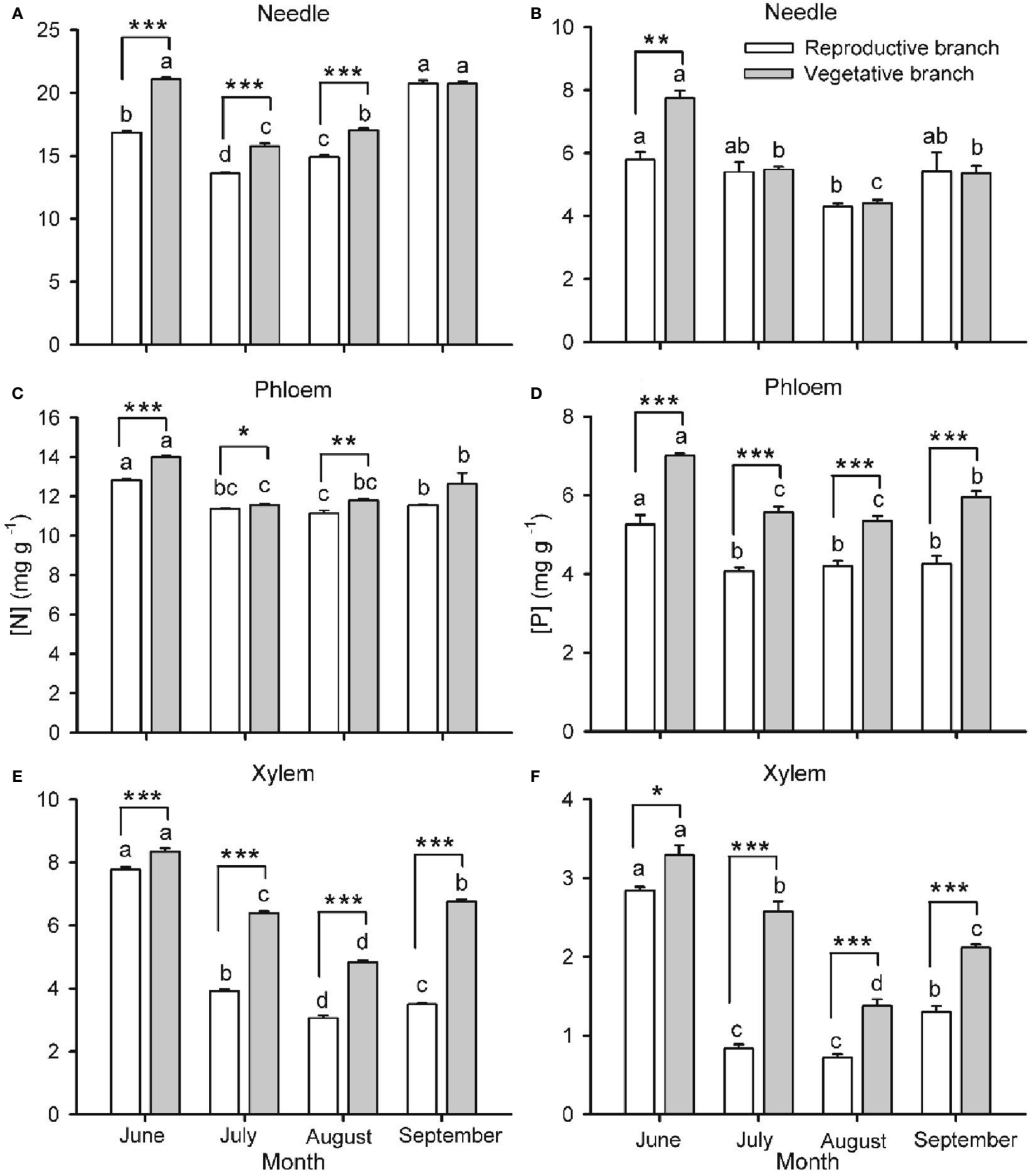
Nitrogen **(**[N]; **A, C, E)** and phosphorus **(**[P]; **B, D, F)** concentrations in needles **(A, B)**, phloem **(C, D)** and xylem **(E, F)** of one-year-old twigs of *Pinus koraiensis* during four sampling times (June, July, August and September, corresponding to DOYs 172, 204, 237 and 258, respectively). Bars and arrows represent the mean and corresponding standard error from five trees, respectively. Asterisks indicate significant differences between branch types for a given harvest time; *, ** and *** indicate significance levels at *P* < 0.05, 0.01 and 0.001, respectively. Different lowercase letters indicate significant differences between harvest times for a given branch type.

**Figure 3:**
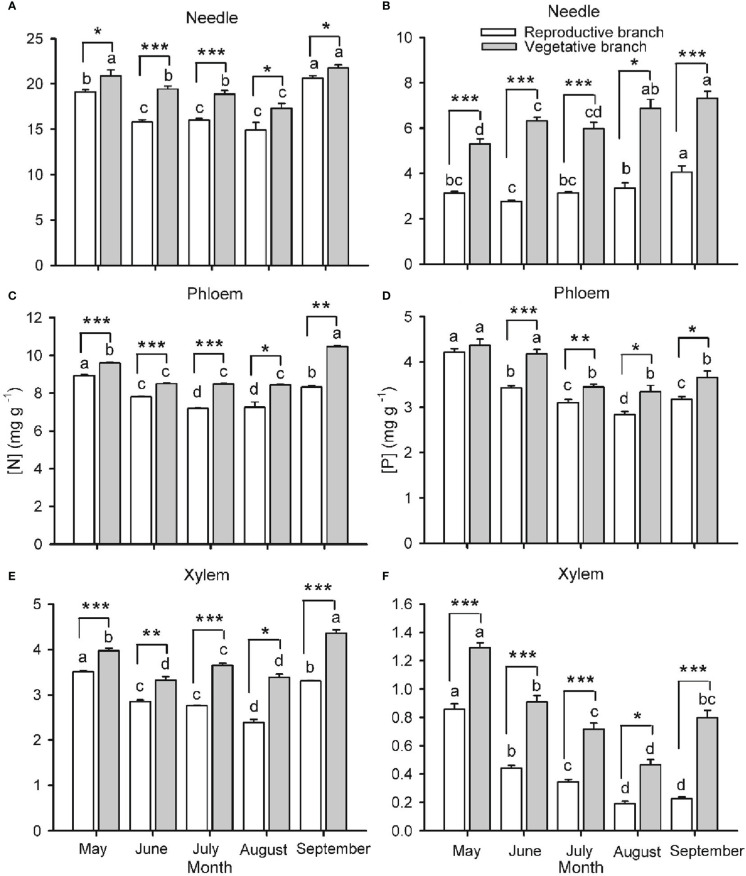
Nitrogen **(**[N]; **A, C, E)** and phosphorus **(**[P]; **B, D, F)** concentrations in needles **(A, B)**, phloem **(C, D)** and xylem **(E, F)** in two-year-old twigs of *Pinus koraiensis* at five sampling times (May, June, July, August and September, corresponding to DOYs 128, 172, 204, 237 and 258, respectively). Bars and arrows represent the mean and corresponding standard error from five trees, respectively. Asterisks indicate significant differences between branch types for a given harvest time; *, ** and *** indicate significance levels at *P* < 0.05, 0.01 and 0.001, respectively. Different lowercase letters indicate significant differences between harvest times for a given branch type.

Branch type (reproductive or vegetative) and harvest time had a significant effect on [N] and [P] in needles, phloem and xylem of both one- (**Figure 2**) and two- (**Figure 3**) years-old twigs, with the interaction between branch type and harvest time being significant in most cases (**Table S1**). Overall, [N] and [P] in needles, phloem and xylem were lower in reproductive branches than in vegetative branches. Among tissues, [N] and [P] were generally highest in needles, lowest in the xylem, and intermediate in the phloem.

Seasonality in [N] and [P] was roughly similar across monitored tissues and for one- and two-year-old twigs (**Figures 2**, **3**, respectively). The concentrations were highest in spring (May-June), decreased as organs matured during summer (July-August), and increased again by the end of the growing season (September), without fully recovering spring values in the case of one-year-old twigs. The only exception to this seasonal behavior was observed for [P] in needles, which increased in two-year-old twigs as the growing season progressed. The seasonality of [N] and [P] was different in reproductive and vegetative branches. For one-year-old twigs, differences in needle [N] and [P] between branch types were higher during early summer than in September, while differences in xylem [N] and [P] were higher in September than in June (**Figure 2**). For two-year-old twigs, xylem and phloem [N] were higher in September than May in vegetative branches and lower in May than September in reproductive branches. Similarly, xylem [P] recovery in September was higher in vegetative than in reproductive branches (**Figure 3**).

**Figure 4:**
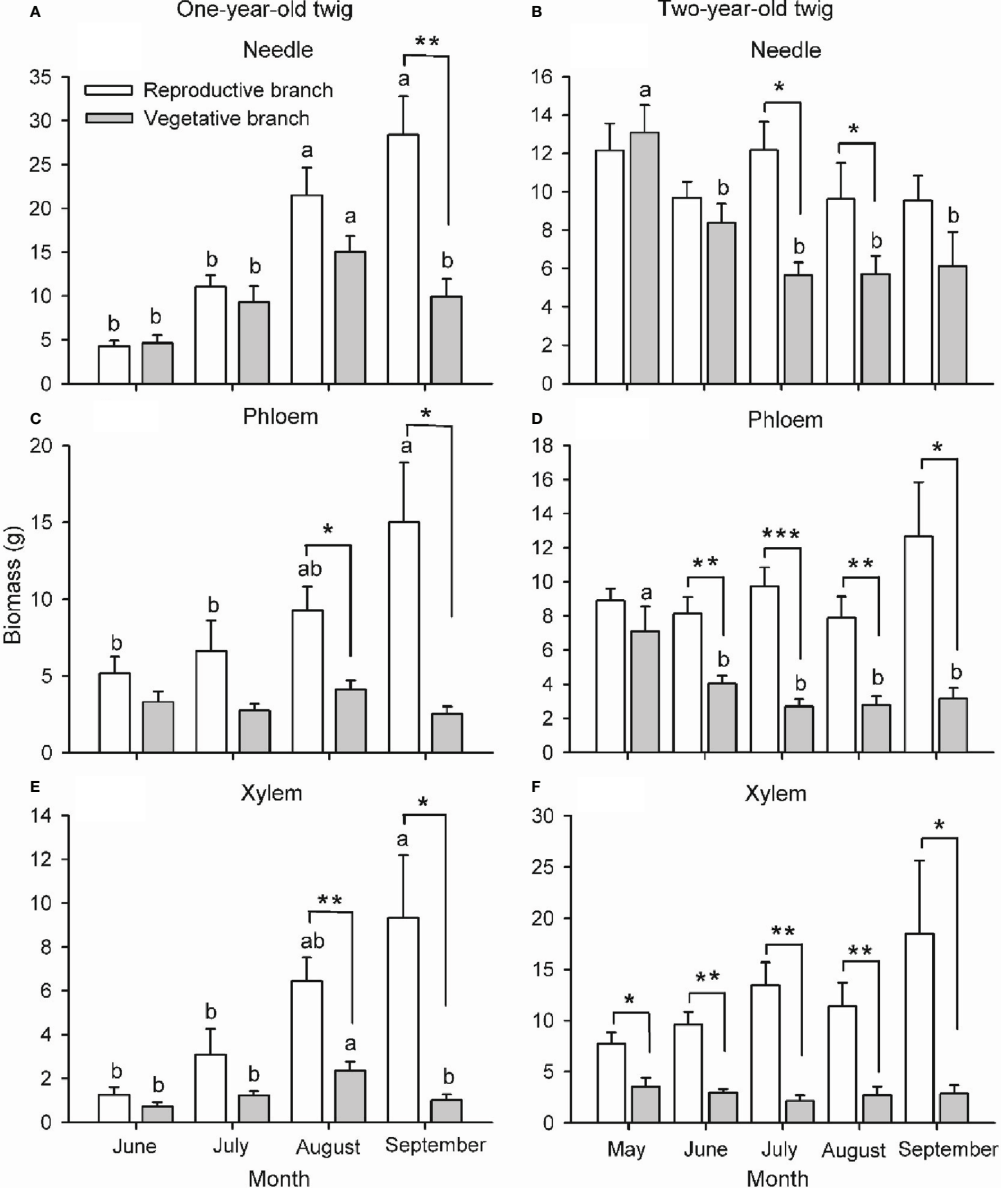
Biomass of needle **(A, B)**, phloem **(C, D)** and xylem **(E, F)** of one- **(A, C, E)** and two-year-old **(B, D, F)** twigs of *Pinus koraiensis* at five sampling times (May, June, July, August and September, corresponding to DOYs 128, 172, 204, 237 and 258, respectively). One-year-old twig biomass was nil in May. Bars and arrows represent the mean and corresponding standard error from five trees, respectively. Asterisks indicate significant differences between branch types for a given harvest time; *, ** and *** indicate significance levels at *P* < 0.05, 0.01 and 0.001, respectively. Different lowercase letters indicate significant differences between harvest times for a given branch type.

**Figure 5:**
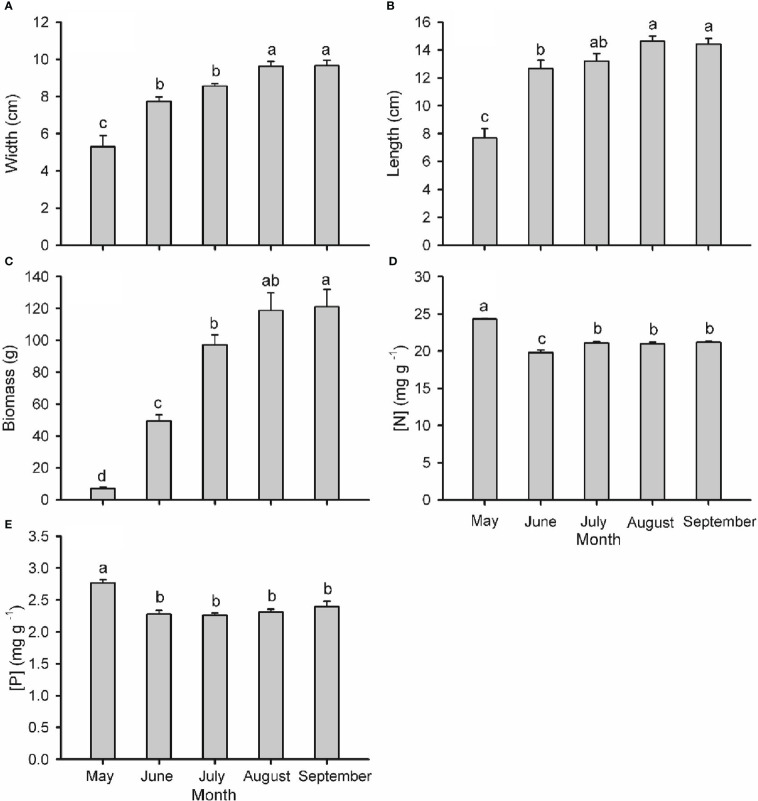
Width **(A)**, length **(B)**, individual biomass **(C)**, nitrogen concentration **(**[N]; **D)** and phosphorus concentration **(**[P]; **E)** in cones of *Pinus koraiensis* at five sampling times (May, June, July, August and September, corresponding to DOYs 128, 172, 204, 237 and 258, respectively). Bars and arrows represent the mean and corresponding standard error from five trees, respectively. Different lowercase letters indicate significant differences among sampling times.

Branch type, harvest time and their interaction had a significant effect on the biomass of needles, phloem and xylem of one-year-old twigs, while these effects were tissue-specific on the biomass of two-year-old twigs (**Table S1**; **Figure 4**). The biomass of all tissues of one- and two-year-old reproductive branches was significantly higher than those of vegetative branches during late-summer (August and/or September), but not in spring (May) except for xylem biomass of two-year-old twigs. In two-year-old twigs of vegetative branches, needle and phloem biomass decreased significantly from spring to summer. The width, length, and biomass of individual cones increased over time from May to August, when growth ceased (**Figure 5**). Cone [N] was highest in May (ca. 24.3 mg g dw^-1^), then decreased significantly in June, and maintained a similar concentration throughout its developmental period after a slight recovery in July (ca. 21.1 mg g dw^-1^). Cone [P] was also highest in May (ca. 2.8 mg g dw^-1^) and maintained a similar concentration of 2.3 mg g dw^-1^ after an initial drop in June.

**Figure 6:**
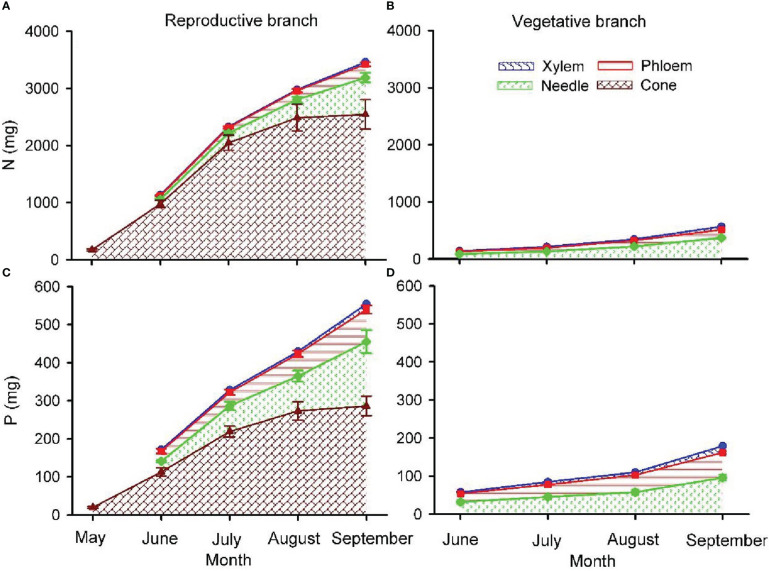
Total nitrogen **(**N; **A, B)** and phosphorus **(**P; **C, D)** content in cone, needle, phloem and xylem of one-year-old twigs of reproductive **(A, C)** and vegetative **(B, D)** branches of *Pinus koraiensis*. The area between lines with different colours indicates the nutrient content in needles, phloem, xylem and cones. Values shown are mean ± SE averaged from five trees. Note that there were no one-year-old twigs in May when branches were sampled.

Due to the relatively small changes in cone [N] and [P] as cones matured, N and P content in cones were primarily determined by biomass dynamics, with the lowest values in May followed by a progressive increase during the growing season (**Figure 6**). At the end of the growing season, in reproductive branches, approximately 73.5% and 51.6% of the N and P content were allocated into cones, 17.6% and 29.4% to needles, 5.7% and 15.3% to the phloem, and 3.2% and 3.7% into the xylem. In vegetative branches, 65.6% and 53.6% of N and P was allocated into needles, 25.8% and 37.9% into the phloem, and 8.6% and 7.5% into the xylem.

**Figure 7:**
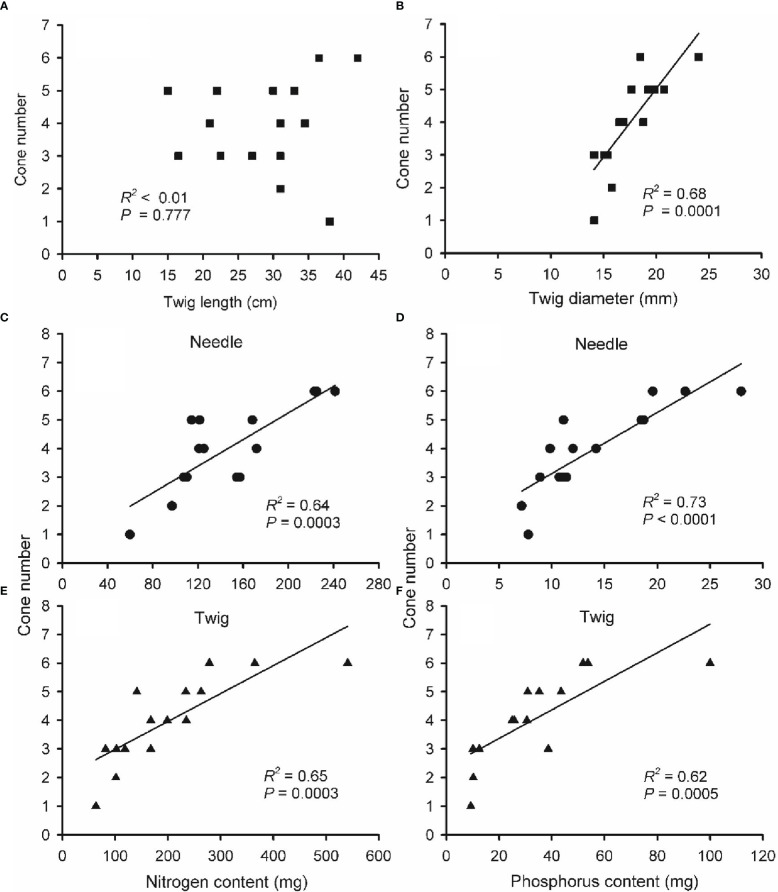
The relationship between cone number and twig length **(A)**, twig diameter **(B)**, nitrogen **(**N; **C, E)** and phosphorus **(**P; **D, F)** content in needles **(C, D)** and twigs **(E, F)** of two-year-old reproductive branches of *Pinus koraiensis*. Data of cone number, twig size, N and P content were obtained from the last sampling campaign (September; five trees × three twigs). Note that some panels show less than 15 points due to overlaying data. Note the different scales for x-axes among panels.

To further understand nutrient effects on cone production, the linear relationships between cone number and end-of-season twig size, N and P content of needle and twigs were explored *via* regression analysis (**Figure 7**). Twigs length was unable to predict cone number (*R^2^* < 0.01, *P* = 0.777). By contrast, cone number was positively correlated with twig diameter, N and P content in needles and twigs of two-year-old twigs (*R^2^* = 0.68, 0.64, 0.73, 0.65 and 0.62, respectively, all *P* < 0.001).

When evaluating the relation between cone number and needle and twig [N] and [P], cone number was positively correlated with [N] and [P] in needles and twigs of two-year-old twigs (*R^2^* = 0.78, 0.90, 0.78 and 0.31, respectively, all *P* < 0.05) (**Figure S1**), indicating the wide range of [N] and [P] in twigs was a significant predictor of cone number.

## 4 Discussion

Large amounts of mineral nutrients and carbohydrates are consumed during a masting event (Kelly, 1994; Allen et al., 2017). The plant life-history theory holds that reproduction and vegetative growth compete for resources, so increased reproductive effort leads to decreased vegetative growth (Wiley and Helliker, 2012). Previous studies support this theory: trees have lower secondary growth in masting than in non-masting years (Vaast et al., 2005; Han et al., 2011), and fruit-bearing branches grow less than branches without fruits (Han et al., 2011; Miyazaki, 2013). However, this is not always the case (Sala et al., 2012; Zhang and Yin, 2019). Contrary to our first hypothesis, results show that the increase in biomass over the last stage of cone maturation was significantly higher in reproductive branches than in vegetative branches (**Figure 4**), denoting that cone development did not limit the growth of reproductive branches. This unexpected observation may be due to two reasons. First, carbohydrates and mineral nutrients needed for cone development come from vegetative branches (Miyazaki et al., 2007; Zhang and Tanabe, 2008; Pasqualotto et al., 2019; Wu et al., 2021), reducing their carbohydrate concentrations and growth. Second, cones could be only formed in the branches storing larger amounts of resources and thus growing (and reproducing) more (Ichie et al., 2005; Yasumura et al., 2006). The latter hypothesis would explain the correlation between cone number and branch dimensions observed in a previous study (Yin et al., 2019) and between cone number and branch diameter, N and P content observed here (**Figure 7**). Consistent with this rationale, previous studies in *Nyssa sylvatica* (Cipollini and Stiles, 1991) and *Rhododendron lapponicum* (Karlsson, 1994) have reported higher nutrient concentrations in reproductive branches before the masting. In the long term, there may be a delayed cost of reproduction, which does not affect the vegetative growth of reproductive branches in the short term, but reduces it during the following seasons (Newell, 1991; Sánchez-Humanes et al., 2011; Sala et al., 2012). Delayed costs of masting in terms of vegetative growth have been observed in *Betula grossa* (Ishihara and Kikuzawa, 2009), *Acer barbinerve* (Zhao et al., 2019) and *P. albicaulis* (Sala et al., 2012).

Because vegetative growth and cone development overlap during the growing season, our second hypothesis anticipated that cone maturation would reduce [N] and [P] in vegetative tissues (needle, phloem and xylem) of reproductive branches to a greater extent than in vegetative ones. Accordingly, [N] and [P] in vegetative tissues of reproductive branches were significantly lower, with the only exception being needle [P] in one-year-old branches (**Figure 2**). A combination of the following factors are likely responsible for this observation: 1) active remobilization of N and P to cones from vegetative tissues, 2) a dilution of N and P as reproductive branches grow bigger (than vegetative branches) and, finally, 3) competition between cones and vegetative tissues for N and P coming from the soil (Han et al., 2008; Tanentzap et al., 2012). Similarly, the high N sink strength of seeds explains N depletion in vegetative structures of fruit-bearing branches in species such as *Fagus sylvatica* and *Taxus baccata* (Han et al., 2011; Nowak-Dyjeta et al., 2017).

In line with our third hypothesis, we expected a progressive depletion of N and P in vegetative tissues as cones mature. In effect, the [N] and [P] in the phloem, xylem and needles were generally lower during mid-summer than in spring and, less so, in late summer (**Figures 2**, **3**). The summer decline in [N] and [P] coincides with the fast shoot and cone growth, as similarly observed in *Quercus ilex* and *Q. faginea* (Silla and Escudero, 2003). These results suggest that N and P invested in shoot and cone growth exceed their root uptake rate, so N and P are being depleted at this time (Dickson, 1989; Han et al., 2008; Nowak-Dyjeta et al., 2017). At the end of the growing season, when the nutrient demand relaxes, N and P pools are expected to replenish. Deciduous species tend to store nutrients in the branches (Tian et al., 2003), trunk (Cooke and Weih, 2005) and roots (Millard and Grelet, 2010), while evergreen species such as Korean pine primarily store nutrients in the foliage and branches (Nambiar and Fife, 1987; Millard et al., 2001; Rodríguez-Calcerrada et al., 2012). Consistently, when cones were mature, and branches had stopped growing in the last sampling date during late summer, [N] and [P] tended to recover in the phloem, xylem and needles, with more marked increases in vegetative branches (**Figure 3**), where the nutrient sink strength for reproductive purposes is absent. Moreover, the complete recovery of end-of-season [N] and [P] to spring values in one-year-old needles (in contrast to the xylem) suggests that current-year xylem replaces the needles in supplying the cones with N and P during maturation (**Figure 2**).

Nitrogen and P are essential components of proteins and nucleic acids required by reproductive organs, which underlies growing evidence showing that N and P depletion after a masting year prevents reproduction during subsequent years (Sala et al., 2012; Han et al., 2014; Yin et al., 2019; Wu et al., 2022). The preferential allocation of nutrients to cone development and seed ripening in one-year-old reproductive branches was evidenced in **Figure 6**: cone N and P content at the end of the growing season accounted for 73.5% and 51.6% of the total nutrient content of current-year branches, respectively. The progressive increase in N and P content was related to a parallel increase in cone size, while [N] and [P] were relatively constant as cones developed. Only in spring (first sampling date), before the initial, fast increase in cone size, were [N] and [P] significantly higher than in the rest of sampling dates. This observation reflects a constant, significant mobilization of nutrients from the soil and storage organs to cones to maintain a stable nutrient status (Nowak-Dyjeta et al., 2017; Wu et al., 2022). Reproductive structures are also nutrient-enriched relative to vegetative biomass in other tree species such as *F. sylvatica* (Han et al., 2011), *Q. ilex* and *Q. faginea* (Alla et al., 2012), and *P. albicaulis* (Sala et al., 2012). Compared to cones, poorer N and P allocation to needles and twig phloem and xylem might hinder flower bud primordial development in subsequent years, as reported in *Fagus crenata* (Han et al., 2008) and *P. albicaulis* (Sala et al., 2012). The branches require a period of replenishment of nutrients after the mast event, which is consistent with rare reproductive events of *P. koraiensis* (Smaill et al., 2011; Sala et al., 2012). Taken together, our results denote that the cone yield of Korean pine would substantially benefit from species-specific designs of fertilization procedures.

## 5 Conclusions

The results of this study evidence that cone growth occurs at the expense of nutrients primarily stored in needles and twigs of reproductive branches of *P. koraiensis*. Needle, phloem and xylem [N] and [P] in one- and two-year-old twigs of reproductive branches were lower than those of vegetative branches. However, twig growth was higher in reproductive than in vegetative branches, suggesting that more vigorous branches tend to produce a larger amount of cones. In fact, the number of cones increased with increasing diameter, N and P content of reproductive branches, with more than half of the N and P in reproductive branches being allocated to the cones. These results suggest that cone development demands a high nutrient cost. Thus, nutrients may need to cumulate to certain levels before another mast event occurs. These results can guide rational fertilization of *P. koraiensis* plantations. Further studies should test whether periodic fertilization promotes nutrient storage and reduces the intervals between masting events.

## Data availability statement

The data analyzed in this study is subject to the following licenses/restrictions: The experimental data used in the study are available from the corresponding author on reasonable request. Requests to access these datasets should be directed to Hailong Shen shenhl-cf@nefu.edu.cn.

## Author contributions

All authors contributed to the study conception and design. PZ and HS conceived and designed the study. DY and JZ collected plant materials and data collection. HW, DY and JR-C analyzed the results for experiments. HW, RS, JR-C and DY contributed to the writing of the manuscript and data analyses. PZ, HS, DY, JR-C and RS revised the manuscript. All authors contributed to the article and approved the submitted version.

## References

[B1] Alla A. Q. Camarero J. J. Maestro-Martínez M. Montserrat-Martí G. (2012). Acorn production is linked to secondary growth but not to declining carbohydrate concentrations in current-year shoots of two oak species. Trees. 26, 841–850. doi: 10.1007/s00468-011-0658-3

[B2] Allen R. B. Hurst J. M. Portier J. Richardson S. J. (2014). Elevation-dependent responses of tree mast seeding to climate change over 45 years. Ecol. Evol. 4, 3525–3537. doi: 10.1002/ece3.1210 25478145 PMC4224528

[B3] Allen R. B. Millard P. Richardson S. J. (2017). A resource centric view of climate and mast seeding in trees. Prog. Botany. 79, 233–268. doi: 10.1007/124_2017_8

[B4] Bogdziewicz M. (2022). How will global change affect plant reproduction? a framework for mast seeding trends. New Phytol. 234, 14–20. doi: 10.1111/nph.17682 34409608

[B5] Bogdziewicz M. Crone E. E. Steele M. A. Zwolak R. (2017). Effects of nitrogen deposition on reproduction in a masting tree: benefits of higher seed production are trumped by negative biotic interactions. J. Ecol. 105, 310–320. doi: 10.1111/1365-2745.12673

[B6] Callahan H. S. Del Fierro K. Patterson A. E. Zafar H. (2008). Impacts of elevated nitrogen inputs on oak reproductive and seed ecology. Global Change Biol. 14, 285–293. doi: 10.1111/j.1365-2486.2007.01483.x

[B7] Cheng C. Mao Z. Jin S. Song G. Sun P. Sun T. (2017). Sensitivity of fruiting for *Pinus koraiensis* to climate change and mechanisms of masting in the original broad-leaved Korean pine forest in north xiaoxing’an mountain, China. Bull. Botanical Res. 37, 118–127.

[B8] Cipollini M. L. Stiles E. W. (1991). Costs of reproduction in *Nyssa sylvatica*: Sexual dimorphism in reproductive frequency and nutrient flux. Oecologia 86, 585–593. doi: 10.1007/BF00318326 28313341

[B9] Congreves K. A. Otchere O. Ferland D. Farzadfar S. Williams S. Arcand M. M. (2021). Nitrogen use efficiency definitions of today and tomorrow. Front. Plant Sci. 12, 637108. doi: 10.3389/fpls.2021.637108 34177975 PMC8220819

[B10] Cook B. I. Wolkovich E. M. Parmesan C. (2012). Divergent responses to spring and winter warming drive community level flowering trends. PNAS 109 (23), 9000–9005. doi: 10.1073/pnas.1118364109 22615406 PMC3384199

[B11] Cooke J. E. Weih M. (2005). Nitrogen storage and seasonal nitrogen cycling in *Populus*: bridging molecular physiology and ecophysiology. New Phytol. 167, 19–30. doi: 10.1111/j.1469-8137.2005.01451.x 15948826

[B12] Dickson R. E. (1989). Carbon and nitrogen allocation in trees. In Annales Des. Sci. forestières. 46, 631–647. doi: 10.1051/forest:198905ART0142

[B13] El Zein R. Bréda N. Gérant D. Zeller B. Maillard P. (2011). Nitrogen sources for current-year shoot growth in 50-year-old sessile oak trees: an *in situ* ^15^N labeling approach. Tree Physiol. 31, 1390–1400. doi: 10.1093/treephys/tpr118 22158010

[B14] Fernández-Martínez M. Pearse I. Sardans J. Sayol F. Koenig W. D. LaMontagne J. M.. (2019). Nutrient scarcity as a selective pressure for mast seeding. Nat. Plants. 5, 1222–1228. doi: 10.1038/s41477-019-0549-y 31792395

[B15] Fernández-Martínez M. Sardans J. Sayol F. LaMontagne J. M. Bogdziewicz M. Collalti A.. (2020). Reply to: Nutrient scarcity cannot cause mast seeding. Nat. Plants. 6, 763–765. doi: 10.1038/s41477-020-0703-6 32572211

[B16] Ghanem G. Ewald A. Zerche S. Hennig F. (2014). Effect of root colonization with *Piriformospora indica* and phosphate availability on the growth and reproductive biology of a *Cyclamen persicum* cultivar. Sci. Hortic-amsterdam. 172, 233–241. doi: 10.1016/j.scienta.2014.04.022

[B17] Han Q. Kabeya D. Hoch G. (2011). Leaf traits, shoot growth and seed production in mature *Fagus sylvatica* trees after 8 years of CO_2_ enrichment. Ann. Bot-london. 107, 1405–1411. doi: 10.1093/aob/mcr082 PMC3101148 21493641

[B18] Han Q. Kabeya D. Iio A. Inagaki Y. Kakubari Y. (2014). Nitrogen storage dynamics are affected by masting events in *Fagus crenata* . Oecologia. 174, 679–687. doi: 10.1007/s00442-013-2824-3 24221082

[B19] Han Q. Kabeya D. Iio A. Kakubari Y. (2008). Masting in *Fagus crenata* and its influence on the nitrogen content and dry mass of winter buds. Tree Physiol. 28, 1269–1276. doi: 10.1093/treephys/28.8.1269 18519258

[B20] Han Q. Kabeya D. Inagaki Y. (2017). Influence of reproduction on nitrogen uptake and allocation to new organs in *Fagus crenata* . Tree Physiol. 37, 1436–1443. doi: 10.1093/treephys/tpx095 28985424

[B21] Ichie T. Kenzo T. Kitahashi Y. Koike T. Nakashizuka T. (2005). How does *Dryobalanops aromatica* supply carbohydrate resources for reproduction in a masting year? Trees. 19, 704–711. doi: 10.1007/s00468-005-0434-3

[B22] Ichie T. Nakagawa M. (2013). Dynamics of mineral nutrient storage for mast reproduction in the tropical emergent tree *Dryobalanops aromatica* . Ecol. Res. 28, 151–158. doi: 10.1007/s11284-011-0836-1

[B23] Ishihara M. I. Kikuzawa K. (2009). Annual and spatial variation in shoot demography associated with masting in *Betula grossa*: comparison between mature trees and saplings. Ann. Bot-london. 104, 1195–1205. doi: 10.1093/aob/mcp217 PMC2766207 19734164

[B24] Jasim A. H. (2013). Effect of foliar fertilizer on growth and yield of seven potato cultivars (*Solanum tuberosom* l.). Sci. Papers-Series B-Horticulture. 57, 77–80.

[B25] Karlsson P. S. (1994). The significance of internal nutrient cycling in branches for growth and reproduction of *Rhododendron lapponicum* . Oikos. 70 (2), 191–200. doi: 10.2307/3545630

[B26] Kelly D. (1994). The evolutionary ecology of mast seeding. Trends Ecol. Evol. 9, 465–470. doi: 10.1016/0169-5347(94)90310-7 21236924

[B27] Kelly D. (2020). Nutrient scarcity cannot cause mast seeding. Nat. Plants. 6, 760–762. doi: 10.1038/s41477-020-0702-7 32572212

[B28] Kelly D. Sork V. L. (2002). Mast seeding in perennial plants: why, how, where? Annu. Rev. Ecol. S. 33, 427–447. doi: 10.1146/annurev.ecolsys.33.020602.095433

[B29] Koenig W. D. Funk K. A. Kraft T. S. Carmen W. J. Knops J. M. H. (2012). Stabilizing selection for within-season flowering phenology confirms pollen limitation in a wind-pollinated tree. J. Ecol. 100, 758–763. doi: 10.2307/41496124

[B30] Koenig W. D. Knops J. M. (2005). The mystery of masting in trees: some trees reproduce synchronously over large areas, with widespread ecological effects, but how and why? Am. Sci. 93, 340–348.

[B31] Law B. Mackowski C. Schoer L. Tweedie T. (2000). Flowering phenology of myrtaceous trees and their relation to climatic, environmental and disturbance variables in northern New South Wales. Austral. Ecol. 25, 160–178. doi: 10.1046/j.1442-9993.2000.01009.x

[B32] LaMontagne J. M. Pearse I. S. Greene D. F. Koenig W. D. (2020). Mast seeding patterns are asynchronous at a continental scale. Nat. Plants. 6, 460–465. doi: 10.1038/s41477-020-0647-x 32341539

[B33] Liao Y. Li C. Ling Y. Hang G. Yan R. (2021). Research progress on woody oil resources in China. J. Chin. Cereals Oils Assoc. 36 (8), 151–159.

[B34] Millard P. Grelet G. A. (2010). Nitrogen storage and remobilization by trees: ecophysiological relevance in a changing world. Tree Physiol. 30, 1083–1095. doi: 10.1093/treephys/tpq042 20551251

[B35] Millard P. Wendler R. Baillie G. (2001). Interspecific defoliation responses of trees depend on sites of winter nitrogen storage. Funct. Ecol. 15, 535–543. doi: 10.1046/j.0269-8463.2001.00541.x

[B36] Miyazaki Y. (2013). Dynamics of internal carbon resources during masting behavior in trees. Ecol. Res. 28, 143–150. doi: 10.1007/s11284-011-0892-6

[B37] Miyazaki Y. Hiura T. Funada R. (2007). Allocation of photo-assimilated ^13^C from reproductive and non-reproductive shoots to fruits in *Styrax obassia* . Plant Spec Biol. 22, 53–57. doi: 10.1111/j.1442-1984.2007.00176.x

[B38] Miyazaki Y. Hiura T. Kato E. Funada R. (2002). Allocation of resources to reproduction in *Styrax obassia* in a masting year. Ann. Bot-london. 89, 767–772. doi: 10.1093/aob/mcf107 PMC4233828 12102532

[B39] Miyazaki Y. Maruyama Y. Chiba Y. Kobayashi M. J. Joseph B. Shimizu K. K.. (2014). Nitrogen as a key regulator of flowering in *Fagus crenata*: understanding the physiological mechanism of masting by gene expression analysis. Ecol. Lett. 17, 1299–1309. doi: 10.1111/ele.12338 25103959

[B40] Munoz N. Guerri J. Legaz F. Primo-Millo E. (1993). Seasonal uptake of ^15^N-nitrate and distribution of absorbed nitrogen in peach trees. Plant Soil. 150, 263–269. doi: 10.1007/BF00013023

[B41] Nakahata R. Naramoto M. Sato M. Mizinaga H. (2021). Multifunctions of fine root phenology in vegetative and reproductive growth in mature beech forest ecosystems. Ecosphere. 12 (10), e03788. doi: 10.1002/ecs2.3788

[B42] Nambiar E. Fife D. (1987). Growth and nutrient retranslocation in needles of radiata pine in relation to nitrogen supply. Ann. Bot-london. 60, 147–156. doi: 10.1093/oxfordjournals.aob.a087431

[B43] Newbery D. M. Chuyong G. B. Zimmermann L. (2006). Mast fruiting of large ectomycorrhizal African rain forest trees: importance of dry season intensity, and the resource-limitation hypothesis. New Phytol. 170, 561–579. doi: 10.1111/j.1469-8137.2006.01691.x 16626477

[B44] Newell E. A. (1991). Direct and delayed costs of reproduction in *Aesculus californica* . J. Ecol. 79 (2), 365–378. doi: 10.2307/2260719

[B45] Nowak-Dyjeta K. Giertych M. Thomas P. Iszkuło G. (2017). Males and females of *Juniperus communis* l. and *Taxus baccata* l. show different seasonal patterns of nitrogen and carbon content in needles. Acta Physiol. Plant 39, 191–198. doi: 10.1007/s11738-017-2489-3

[B46] Pasqualotto G. Carraro V. De Gregorio T. Huerta E. S. Anfodillo T. (2019). Girdling of fruit-bearing branches of *Corylus avellana* reduces seed mass while defoliation does not. Sci. Hortic-amsterdam. 255, 37–43. doi: 10.1016/j.scienta.2019.05.016

[B47] Pearse I. S. Koenig W. D. Kelly D. (2016). Mechanisms of mast seeding: resources, weather, cues, and selection. New Phytol. 212, 546–562. doi: 10.1111/nph.14114 27477130

[B48] Pérez-Ramos I. M. Padilla-Díaz C. M. Koenig W. D. Marañón T. (2015). Environmental drivers of mast-seeding in Mediterranean oak species: does leaf habit matter? J. Ecol. 103 (3), 691–700. doi: 10.1111/1365-2745.12400

[B49] Proe M. Millard P. (1994). Relationships between nutrient supply, nitrogen partitioning and growth in young sitka spruce (*Picea sitchensis*). Tree Physiol. 14, 75–88. doi: 10.1093/treephys/14.1.75 14967635

[B50] Rapp M. Santa-Regina I. Rico M. Gallego H. A. (1999). Biomass, nutrient content, litterfall and nutrient return to the soil in Mediterranean oak forests. For. Ecol. Managt. 119, 39–49. doi: 10.1016/S0378-1127(98)00508-8

[B51] Rodríguez-Calcerrada J. Limousin J. M. Martin-StPaul N. K. Jaeger C. Rambal S. (2012). Gas exchange and leaf aging in an evergreen oak: causes and consequences for leaf carbon balance and canopy respiration. Tree Physiol. 32, 464–477. doi: 10.1093/treephys/tps020 22491489

[B52] Roland C. A. Schmidt J. H. Johnstone J. F. (2014). Climate sensitivity of reproduction in a mast-seeding boreal conifer across its distributional range from lowland to treeline forests. Oecologia. 174, 665–677. doi: 10.1007/s00442-013-2821-6 24213628

[B53] Sala A. Hopping K. McIntire E. J. Delzon S. Crone E. E. (2012). Masting in whitebark pine (*Pinus albicaulis*) depletes stored nutrients. New Phytol. 196, 189–199. doi: 10.1111/j.1469-8137.2012.04257.x 22889129

[B54] Sánchez-Humanes B. Sork V. L. Espelta J. M. (2011). Trade-offs between vegetative growth and acorn production in *Quercus lobata* during a mast year: the relevance of crop size and hierarchical level within the canopy. Oecologia. 166, 101–110. doi: 10.1007/s00442-010-1819-6 21049300 PMC3074067

[B55] Satake A. Bjørnstad O. N. (2008). A resource budget model to explain intraspecific variation in mast reproductive dynamics. Ecolog Res. 23, 3–10. doi: 10.1007/s11284-007-0397-5

[B56] Shen H. L. (2003). Korean Pine as a nut production species in china-present situation and future development. Acta Hortic. 620, 87–91.

[B57] Shen J. Yuan X. Li M. Yu F. Wang X. Liu L.. (2019). Effects of soil temperature and moisture on nitrogen and phosphorus contents in *Picea balfouriana* seedlings. Scientia Silvae Sinicae. 55, 31–41. doi: 10.11707/j.1001-7488.20190404

[B58] Silla F. Escudero A. (2003). Uptake, demand and internal cycling of nitrogen in saplings of Mediterranean quercus species. Oecologia. 136, 28–36. doi: 10.1007/s00442-003-1232-5 12820065

[B59] Smaill S. J. Clinton P. W. Allen R. B. Davis M. R. (2011). Climate cues and resources interact to determine seed production by a masting species. J. Ecol. 99, 870–877. doi: 10.1111/j.1365-2745.2011.01803.x

[B60] Smith H. M. Samach A. (2013). Constraints to obtaining consistent annual yields in perennial tree crops. I: Heavy fruit load dominates over vegetative growth. Plant Sci. 207, 158–167. doi: 10.1016/j.plantsci.2013.02.014 23602111

[B61] Sork V. L. Bramble J. Sexton O. (1993). Ecology of mast-fruiting in three species of north American deciduous oaks. Ecology 74, 528–541. doi: 10.2307/1939313

[B62] Tanentzap A. J. Lee W. G. Coomes D. A. (2012). Soil nutrient supply modulates temperature-induction cues in mast-seeding grasses. Ecology. 93, 462–469. doi: 10.1890/11-1750.1 22624201

[B63] Tian W. M. Wu J. L. Hao B. Z. Hu Z. H. (2003). Vegetative storage proteins in the tropical tree *Swietenia macrophylla*: seasonal fluctuation in relation to a fundamental role in the regulation of tree growth. Can. J. Bot. 81, 492–500. doi: 10.1139/b03-045

[B64] Turner J. Lambert M. J. Humphreys F. (2002). Continuing growth response to phosphate fertilizers by a *Pinus radiata* plantation over fifty years. For. Sci. 48, 556–568. doi: 10.1093/forestscience/48.3.556

[B65] Vaast P. Angrand J. Franck N. Dauzat J. Génard M. (2005). Fruit load and branch ring-barking affect carbon allocation and photosynthesis of leaf and fruit of *Coffea arabica* in the field. Tree Physiol. 25, 753–760. doi: 10.1093/treephys/25.6.753 15805095

[B66] Van den Driessche R. (1985). Late-season fertilization, mineral nutrient reserves, and retranslocation in planted Douglas-fir (*Pseudotsuga menziesii* (Mirb.) Franco) seedlings. For. Sci. 31, 485–496. doi: 10.1093/forestscience/31.2.485

[B67] Venner S. Siberchicot A. Pélisson P.-F. Schermer E. Bel-Venner M.-C. Nicolas M.. (2016). Fruiting strategies of perennial plants: a resource budget model to couple mast seeding to pollination efficiency and resource allocation strategies. Am. Nat. 188 (1), 66–75. doi: 10.1086/686684 27322122

[B68] Wang Z. Y. Chen X. Q. (2004). Functional evaluation for effective compositions in seed oil of Korean pine. J. Forestry Res. 15 (3), 215–217. doi: 10.1007/BF02911028

[B69] Wang W. J. Watanabe Y. Endo I. Kitaoka S. Koike T. (2006). Seasonal changes in the photosynthetic capacity of cones on a larch (*Larix kaempferi*) canopy. Photosynthetica. 44, 345–348. doi: 10.1007/s11099-006-0034-5

[B70] Wiley E. Helliker B. (2012). A re-evaluation of carbon storage in trees lends greater support for carbon limitation to growth. New Phytol. 195, 285–289. doi: 10.1111/j.1469-8137.2012.04180.x 22568553

[B71] Wu H. Yin D. Rodríguez-Calcerrada J. Zhang J. Gil L. Zhang P.. (2022). Cone-bearing effects on photosynthetic traits do not change with needle age in *Pinus koraiensis* trees. New Forest 53, 607–626. doi: 10.1007/s11056-021-09874-x

[B72] Wu H. Yin D. Salomón R. L. Rodríguez-Calcerrada J. Zhang J. Zhang P.. (2021). Cone-bearing branches of *Pinus koraiensis* are not carbon autonomous during cone development. Forests 12, 1257. doi: 10.3390/f12091257

[B73] Xie K. Y. Miles E. A. Calder P. C. (2016). A review of the potential health benefits of pine nut oil and its characteristic fatty acid pinolenic acid. J. Funct. Foods. 23, 464–473. doi: 10.1016/j.jff.2016.03.003

[B74] Yamauchi A. (1996). Theory of mast reproduction in plants: storage-size dependent strategy. Evolution. 50, 1795–1807. doi: 10.2307/2410737 28565602

[B75] Yasumura Y. Hikosaka K. Hirose T. (2006). Resource allocation to vegetative and reproductive growth in relation to mast seeding in *Fagus crenata* . For. Ecol. Manage. 229, 228–233. doi: 10.1016/j.foreco.2006.04.003

[B76] Yin D. Wu H. Zhang J. Ge W. Zhou Z. Shen H. (2019). Effects of girdling and defoliation on the growth of female cones and branches and nutrient content in different tissues and organs of *Pinus koraiensis* . Chin. J. Appl. Ecol. 30, 3671–3680. doi: 10.13287/j.1001-9332.201911.011 31833679

[B77] Zhang P. Liu C. Shen H. (2017). Edible nut pine trees and their utilization worldwide. World Forestry Res. 30 (1), 12–17. doi: 10.13348/j.cnki.sjlyyj.2017.0004.y

[B78] Zhang C. Tanabe K. (2008). Partitioning of ^13^C-photosynthates from different current shoots neighboring with fruiting spur in later-maturing Japanese pear during the period of rapid fruit growth. Sci. Hortic-amsterdam. 117, 142–150. doi: 10.1016/j.scienta.2008.03.034

[B79] Zhang J. Yin D. (2019). Effects of female cone development on the vegetative growth and biomass accumulation of shoots and needles of *Pinus koraiensis* . Chin. J. Ecol. 38, 1646–1652. doi: 10.13292/j.1000-4890.201906.016

[B80] Zhao H. Song Z. Xu M. Huang Y. Zhang X. Wang J. (2019). Delayed effects of reproductive costs in dioecious species *Acer barbinerve* . J. Beijing Forestry University. 41, 84–93. doi: 10.13332/j.1000-1522.20180360

