# Supplementary material for: Large investment of stored nitrogen and phosphorus in female cones is consistent with infrequent reproduction events of Pinus koraiensis, a high value woody oil crop in Northeast Asia
Source: Front Plant Sci. 2023 Jan 12;13:1084043. doi: 10.3389/fpls.2022.1084043 (PMC9878279; doi:10.3389/fpls.2022.1084043)
Supplement: Supplementary file 1 [file DataSheet_1.docx]

Table. S1 Effect of harvest time (HT, May, June, July, August and September), branch type (BT, reproductive vs vegetative branches) and their interaction (HT x BT) on biomass, nitrogen and phosphorus concentrations ([N] and [P]) in needles, phloem and xylem of one- and two-year-old twigs of *Pinus koraiensis*.

|  | One-year-old branch | | | | | | | | | | Two-year-old branch | | | | | | | | | |
| --- | --- | --- | --- | --- | --- | --- | --- | --- | --- | --- | --- | --- | --- | --- | --- | --- | --- | --- | --- | --- |
|  | Biomass | | | [N] | | | | [P] | | | Biomass | | | [N] | | | [P] | | | |
|  | Needle | Phloem | Xylem | Needle | Phloem | Xylem | Needle | | Phloem | Xylem | Needle | Phloem | Xylem | Needle | Phloem | Xylem | | Needle | Phloem | Xylem |
| HT | **23.32**  ******* | **4.53**  ****** | **7.92**  ****** | **681.5**  ******* | **29.29**  ******* | **5926.52**  ******* | **23.12**  ******* | | **33.7**  ******* | **291.95**  ******* | **7.5**  ******* | 2.22  ns | 1.63  ns | **41.21**  ******* | **127.1**  ******* | **116.18**  ******* | | **12.5**  ******* | **49.81**  ******* | **130.79**  ******* |
| BT | **20.47**  ******* | **36.01**  ******* | **28.71**  ******* | **402.5**  ******* | **20.26**  ******* | **7886.29**  ******* | **6.53**  ***** | | **193.26**  ******* | **522.42**  ******* | **6.12**  ***** | **60.32**  ******* | **45.95**  ******* | **68.02**  ******* | **385.91**  ******* | **501.3**  ******* | | **437.11**  ******* | **54.64**  ******* | **373.63**  ******* |
| HT x BT | **8.44**  ******* | **5.59**  ******* | **6.11**  ******* | **67.21**  ******* | 1.83  ns | **620.83**  ******* | **5.43**  ****** | | 1.52  ns | **46.91**  ******* | 2.49  ns | **3.39**  ***** | 2.06  ns | 2.24  ns | **19.38**  ******* | **13.55**  ******* | | **3.12**  ***** | 2.59  ns | **5.02**  ****** |

Linear mixed models were adjusted, with the tree considered a random factor. F-values are shown. Significant (*P* < 0.05) results are in bold. ns denotes a not significant effect; *, ** and *** indicate significance levels at P< 0.05, 0.01 and 0.001, respectively.


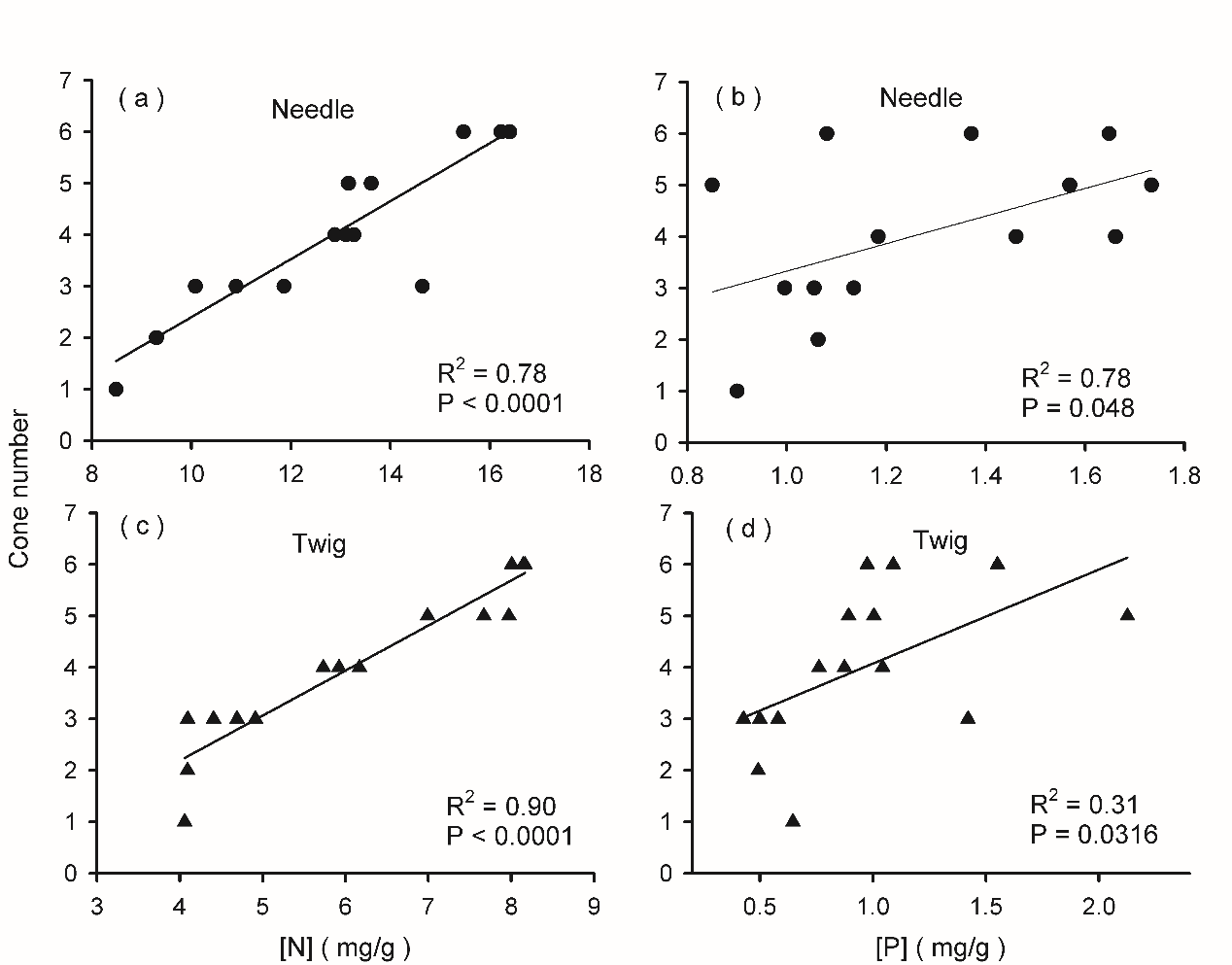


Fig. S1 The relationship between cone number and nitrogen ([N]; a, c) and phosphorus (b, d) concentration in needles ([P]; a, b) and twigs (c, d) of two-year-old reproductive branches of *Pinus koraiensis*. Data of cone number, [N] and [P] were obtained from the last sampling campaign (September; five trees × three twigs). Note that some panels show less than 15 points due to overlaying data. Note the different scales for x-axes among panels.
